# PD-L1 expression and CD8+ tumor-infiltrating lymphocytes are associated with ALK rearrangement and clinicopathological features in inflammatory myofibroblastic tumors

**DOI:** 10.18632/oncotarget.20948

**Published:** 2017-09-15

**Authors:** Yoon Jin Cha, Hyo Sup Shim

**Affiliations:** ^1^ Department of Pathology, Severance Hospital, Yonsei University College of Medicine, Seoul, Korea

**Keywords:** myofibroblastic tumor, inflammatory, anaplastic lymphoma kinase, PD-L1, lymphocyte, Pathology Section

## Abstract

**Background:**

Inflammatory myofibroblastic tumors (IMTs) are rare mesenchymal neoplasms that are composed of myofibroblastic cells accompanied by inflammatory infiltrate. We investigated the immune profiles of IMTs, including PD-L1 expression and proportion of CD8+ tumor-infiltrating lymphocytes (TILs), as well as its clinicopathological characteristics according to *ALK* gene rearrangementstatus.

**Methods:**

Twenty-eight IMTs from 25 patients were retrieved from our pathology files (2005–2015), and their clinicopathological parameters and outcomes were analyzed. Immunohistochemistry (IHC) was performed using whole-tissue sections to detect PD-L1 and CD8 expression, and fluorescent in situ hybridization (FISH) analysis and IHC were performed using tissue microarrays to identify rearrangements in the *ALK, ROS1*, and *RET* genes.

**Results:**

*ALK* rearrangement was observed in 11 cases (44.0%), and all cases exhibited diffuse cytoplasmic *ALK* expression during IHC. *ROS1* or *RET* rearrangement was not detected using IHC or FISH. IMTs harboring *ALK* rearrangement (*ALK*-positive) were located in the lungs (*n* = 7), genitourinary tract (*n* = 2), and mesentery (*n* = 1). The mean patient age was 33.2 years for *ALK*-positive IMTs and 53.1 years for *ALK*-negative IMTs. All patients with *ALK*-positive IMTs survived without recurrence or metastasis. IMTs with metastasis and/or recurrence were *ALK*-negative and exhibited elevated PD-L1 expression (positive tumor cells: 70.0% *vs*. 21.3%, *P* = 0.023; H-score: 107.5 *vs*. 26.3, *P* = 0.005). In addition, *ALK*-negative IMTs had a more CD8+ TILs, compared to *ALK*-positive IMTs (23.3% *vs*. 8.9%, *P* = 0.027).

**Conclusion:**

*ALK*-positive IMTs are characterized by younger age, well-defined margins, frequent involvement of the lung, and fewer CD8+ TILs. Greater PD-L1 expression was observed in IMTs with tumor necrosis and metastasis/recurrence, which were also negative for *ALK* rearrangement. These results suggest that immune checkpoint inhibitors may be a novel option for treating patients with advanced IMT.

## INTRODUCTION

Inflammatory myofibroblastic tumors (IMT) are rare but distinctive mesenchymal neoplasms that are composed of myofibroblastic cells accompanied by inflammatory infiltrate. These tumors can occur at any anatomical site, but typically involve the lung, soft tissue, and viscera of children and young adults [1]. Approximately half of IMTs harbor *ALK* gene rearrangement [2], and small subsets harbor kinase fusions, such as *ROS1, PDGFRβ,* and *RET* [3, 4]. These kinase fusions could be therapeutically targeted using tyrosine kinase inhibitors, as patients with IMTs harboring *ALK* rearrangements (*ALK*-positive) experienced sustained partial response to an *ALK* inhibitor (crizotinib) [5]. Immune checkpoint inhibitors have also recently provided substantial therapeutic activity in various tumors that express PD-L1 or have rich inflammatory components, such as malignant melanoma [6] and Hodgkin lymphoma [7]. The success of immunotherapy and interest in immune checkpoint inhibitors has led to research regarding PD-L1 and tumor-infiltrating lymphocytes (TILs) in various tumors. As IMTs are characterized by TILs, we hypothesized that the immune checkpoint pathway would be involved in a subset of IMTs, which could have therapeutic implications for patients with advanced IMT. The present study aimed to evaluate PD-L1 expression and CD8+ TILs in IMTs, as well as the clinicopathological characteristics of IMTs according to *ALK* status.

## RESULTS

### Patient characteristics

Twenty-eight IMTs from 25 patients were examined, and their clinicopathological profiles are summarized in Table 1. The patients included 13 men and 12 women, with a mean age of 44.3 years (range: 0-76 years). One patient died because of the disease, 3 patients experienced local recurrence and/or metastasis, and 4 patients were lost to follow-up. The lung was the most common site of involvement (*n* = 8), and the genitourinary tract (*n* = 5) was the most common extrapulmonary site. Most patients (*n* = 23) underwent surgical resection and 2 patients were followed-up after the biopsy without further treatment. One patient received chemotherapy because of metastasis and 1 patient received radiotherapy because of local recurrence.

**Table 1:** Clinicopathological profiles of the 25 patients with inflammatory myofibroblastic tumors

Case No.	Age (years)	Sex	Site	Size (cm)	ALK IHC	ALK FISH	Recurrence/ metastasis	Treatment	FU period (mo)	Patient outcome
1	0	M	Mesentery	8.7	+	+	No	Surgical resection	52.5	NED
2	12	M	Lung	3.5	+	+	No	Surgical resection	69.5	NED
3	22	M	Lung	0.6	+	+	No	Surgical resection	108.9	NED
4	24	F	Lung	7.5	+	+	No	Surgical resection	53.8	NED
5	27	F	Lung	2.7	+	+	No	Surgical resection	21.8	NED
6	36	M	Lung	6.0	+	+	No	Surgical resection	6.9	NED
7	38	M	Bladder	2.4	+	+	No	Surgical resection	18.2	NED
8	45	F	Lung	1.9	+	+	No	Surgical resection	0.9	NED
9	47	M	Lung	1.7	+	+	No	Surgical resection	101.2	NED
10	54	F	Stomach	1.5	+	+	No	Surgical resection		
11	60	M	Ureter	1.3	+	+	No	Surgical resection	91.5	NED
12	22	M	Mediastinum	11.0	-	-	No	Surgical resection	5.6	DUD
13	28	F	Salivary gland	1.7	-	-	No	Surgical resection	83.6	NED
14	40	M	Mesentery	6.7	-	-	No	Surgical resection	6.2	NA
15	44	F	Kidney	5.3	-	-	No	Surgical resection	118.3	NA
16	49	M	Maxillary sinus	4.2	-	-	Yes	Surgical resection (incomplete)	11.9	DAD
16a	49	M	Lung	Upto 9.5	-	-	Metastasis, multiple (8mo)	Surgical resection and CTx		
16b	49	M	Maxillary sinus	6.1	-	-	Local recurrence (8mo)	Surgical resection (incomplete) and CTx		
17	49	F	Uterus	7.8	-	-	No	Surgical resection		
18	51	F	Kidney	2.5	-	-	No	Surgical resection	37.8	NED
19	55	M	Kidney	2.5 and 2.3	-	-	Yes	Bx & FU	17.2	AWD
19a	55	M	Lung	3.0	-	-	Metastasis, multiple (synchronously detected)	Surgical resection	17.2	AWD
20	57	M	Mediastinum	8.5	-	-	No	Surgical resection	18.5	NED
21	60	M	Lung	0.8	-	-	No	Surgical resection	63.3	NED
22	69	M	Retroperitoneum	13.6	-	-	No	Bx & FU	27.8	AWD
23	70	F	Maxillary sinus	4.7	-	-	Local recurrence (1.0 mo)	Surgical resection RTx after local recurrence	35.7	NA
24	72	F	Retroperitoneum	5.8	-	-	No	Bx & FU	4.8	NA
25	76	F	Mesentery	4.0	-	-	No	Surgical resection	58.7	NED

IHC, immunohistochemical staining; FISH, fluorescent in situ hybridization; mo, month; M, male; F, female; +, positive; -, negative; postop., postoperative; Bx, biopsy; FU, follow-up; CTx, chemotherapy; RTx, radiotherapy; NA, not available; NED, no evidence of disease; AWD, alive with disease; DOD, died of disease; DUD, died of unrelated cause

Case 16 had metastatic (16a) and recurrent (16b) tumors.

Case 19 had synchronously detected lung metastasis (19a).

### Clinicopathological findings according to *ALK* status

Eleven cases (42.3%) had *ALK* rearrangement, and expression of ALK during the IHC was associated with the *ALK* break-apart FISH results. All *ALK*-positive IMTs exhibited diffuse granular cytoplasmic expression during the IHC (Figure 2A) and split signals during the FISH (Figure 2B). All cases were negative for ROS1 and RET during the IHC and subsequent FISH analysis.

**Figure 1:**
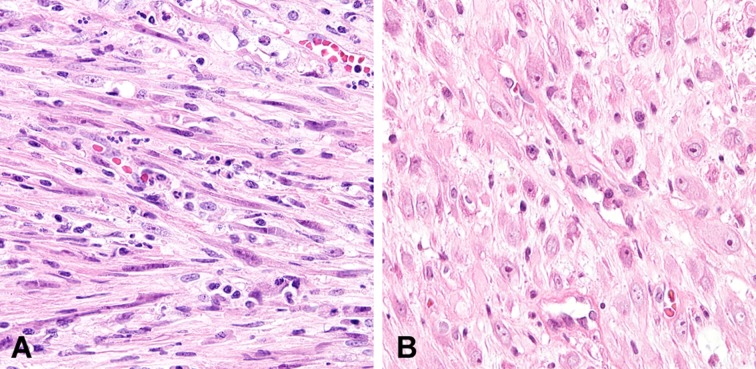
Nuclear features of inflammatory myofibroblastic tumors **A.** Spindled/elongated cells are arranged in loose fascicles. **B.** There is a proliferation of plump epithelioid cells with prominent nucleoli that resemble ganglion cells.

**Figure 2:**
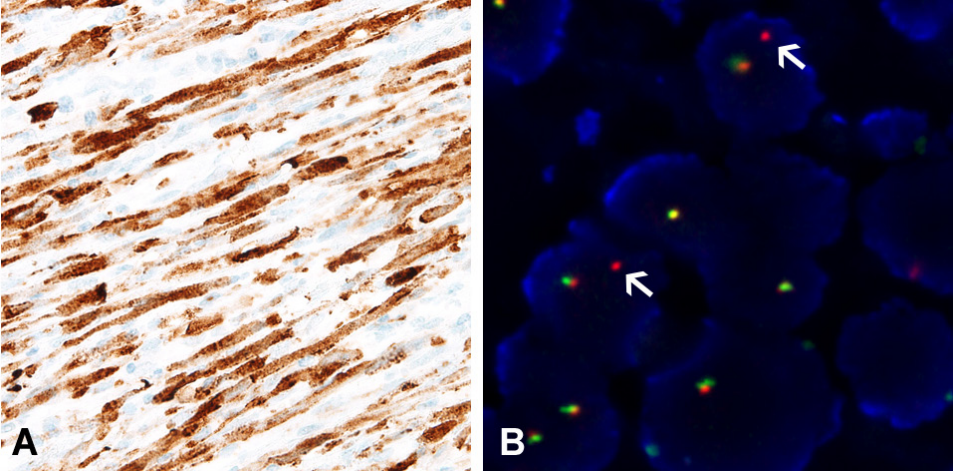
Confirmation of ALK rearrangement using immunohistochemistry and fluorescent *in situ* hybridization analysis **A.** All *ALK*-rearranged inflammatory myofibroblastic tumors display diffusely strong granular cytoplasmic expression of ALK during immunohistochemistry. **B.** Split signals confirm the presence of *ALK* rearrangement.

**Figure 3:**
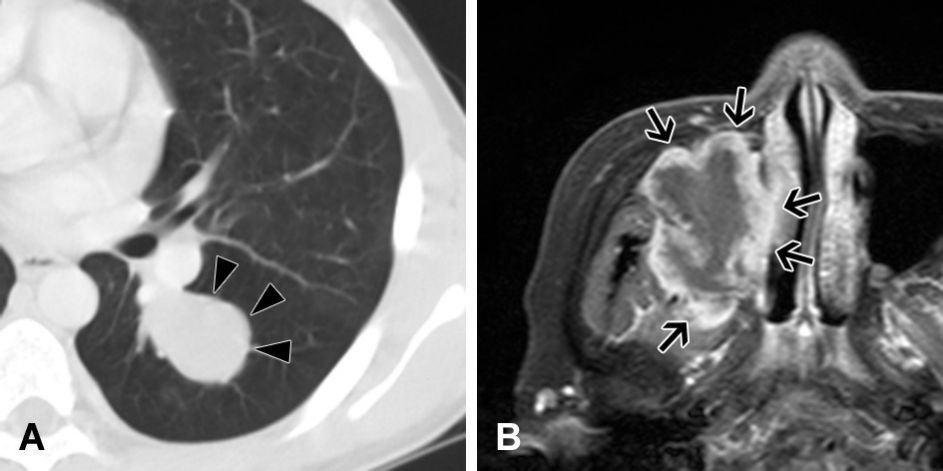
Imaging of inflammatory myofibroblastic tumors **A.** Chest computed tomography reveals a tumor with smooth and well-defined margins in the lung parenchyma of a 12-year-old boy (Case 2). This tumor is positive for *ALK* rearrangement. **B.** Head magnetic resonance imaging reveals a lobulated irregular tumor arising in the sinusoidal space of a 70-year-old woman (Case 24). This tumor is negative for *ALK* rearrangement. Resection was incomplete during the first surgery, and the patient experienced local recurrence after 1 month.

A comparison of the IMTs’ clinicopathological features according to *ALK* rearrangement status is shown in Table 2. Compared to *ALK*-negative IMTs, *ALK*-positive IMTs occurred at a younger age, were mostly located in the lungs, and were more circumscribed (Figure 3). All patients with *ALK-*positive IMTs underwent surgical resection and none of the patients experienced metastasis, recurrence, or death. There were no statistically significant differences in OS or DFS according to *ALK* status. However, *ALK*-positive IMTs tended to have superior OS and DFS (Supplementary Figure 1).

**Table 2:** Clinicopathological and histological features of primary inflammatory myofibroblastic tumors according to *ALK* status

	ALK (−) (*N* = 14)	ALK (+) (*N* = 11)	*P* - value
***Clinicopathological parameters***			
Sex (male) (%)	6 (42.9)	7 (63.6)	0.302^1^
Age (years, mean±SD)	53.0±16.1	33.2±18.2	**0.008**^2^
Tumor size (cm, mean±SD)	5.7±3.6	3.4±2.7	0.106^2^
Pulmonary location (%)	1 (7.1)	7 (63.6)	**0.007**^3^
Well-defined margins (%)	5 (35.7)	9 (81.8)	**0.042**^1^
Adjuvant treatment (%)	2 (14.3)	0 (0.0)	0.487^3^
Death (%)	1 (7.1)	0 (0.0)	**0.043**^3^
Overall survival (months, mean±SD)	35.2±34.5	50.7±38.2	0.297^2^
Metastasis/recurrence (%)	3 (21.4)	0 (0.0)	0.230^3^
Disease-free survival (months, mean±SD)	32.2±36.0	50.7±38.2	0.227^2^
***Histomorphological parameters***			
Morphology of myofibroblastic cells			0.604^3^
Spindle (%)	11 (78.6)	10 (90.9)	
Plump, epithelioid (%)	3 (21.4)	1 (9.1)	
Nuclear atypia of myofibroblastic cells (%)	6 (46.2)	5 (45.5)	1.000^3^
Prominent nucleoli (%)	1 (7.1)	5 (45.5)	0.061^3^
Necrosis (%)	2 (14.3)	3 (27.3)	0.623^3^
PD-L1 positivity	4 (36.4)	6 (54.5)	0.392^3^
CD8+ TIL (%)	23.3±17.8	8.9±6.7	**0.027**^2^

ALK (-), Negative for *ALK* rearrangement; ALK (+), Positive for *ALK* rearrangement; SD, standard deviation; TIL, tumor infiltrating lymphocytes

^1^Chi-square test, ^2^T-test, ^3^Fisher's exact test

Significant values in bold.

### Results from IHC for PD-L1 and CD8

**Figure 4:**
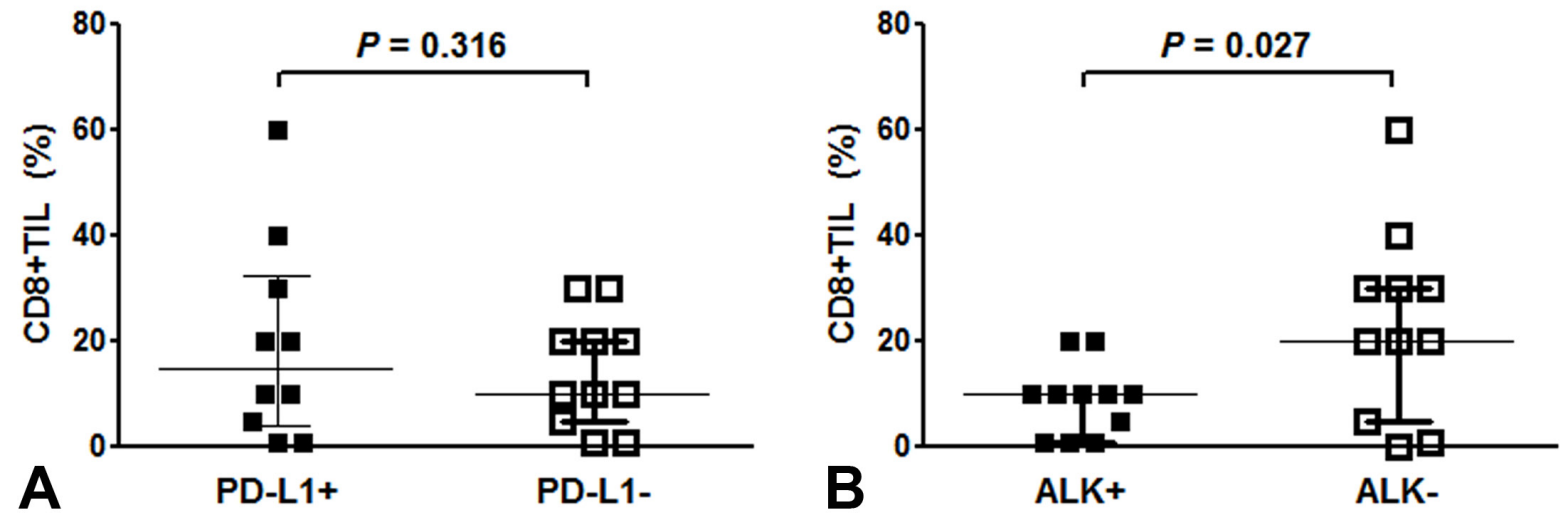
CD8-positive tumor infiltrating lymphocytes (TILs) in inflammatory myofibroblastic tumors (IMTs) based on PD-L1 **A.** and *ALK* **B.** status. The proportion of CD8-positive TILs was slightly higher in PD-L1-positive IMTs, compared to PD-L1-negative IMTs (19.7 ± 19.0% *vs*. 13.1 ± 10.8%, *P* = 0.316) **A.**. *ALK*-negative IMTs had greater CD8-positive TIL infiltration, compared to *ALK*-positive IMTs (mean: 23.3% *vs*. 8.9% *P* = 0.027) **B.**.

PD-L1 positivity was observed in 10 of the 22 available cases (45.5%). There were no differences in the clinicopathological features according to the PD-L1 results. In addition, there were no statistically significant differences in OS or DFS according to PD-L1 status, although PD-L1-positive IMTs tended to have poorer OS and DFS (Supplementary Figure 1). The rate of PD-L1 positivity was not significantly different in the *ALK*-positive and *ALK*-negative tumors (*ALK*-positive: 36.4% *vs*. *ALK*-negative: 54.5%, *P* = 0.392) (Table 2). The average proportion of CD8+ TILs was 18.5% among all IMTs. The proportion of CD8+ TILs was slightly higher in PD-L1-positive IMTs, compared to PD-L1-negative IMTs, although the difference was not significant (19.7 ± 19.0% *vs*. 13.1 ± 10.8%, *P* = 0.316) (Figure 4A). *ALK*-negative IMTs had more CD8+ TILs, compared to *ALK*-positive IMTs (23.3% *vs*. 8.9% *P* = 0.027) (Figure 4B).

**Figure 5:**
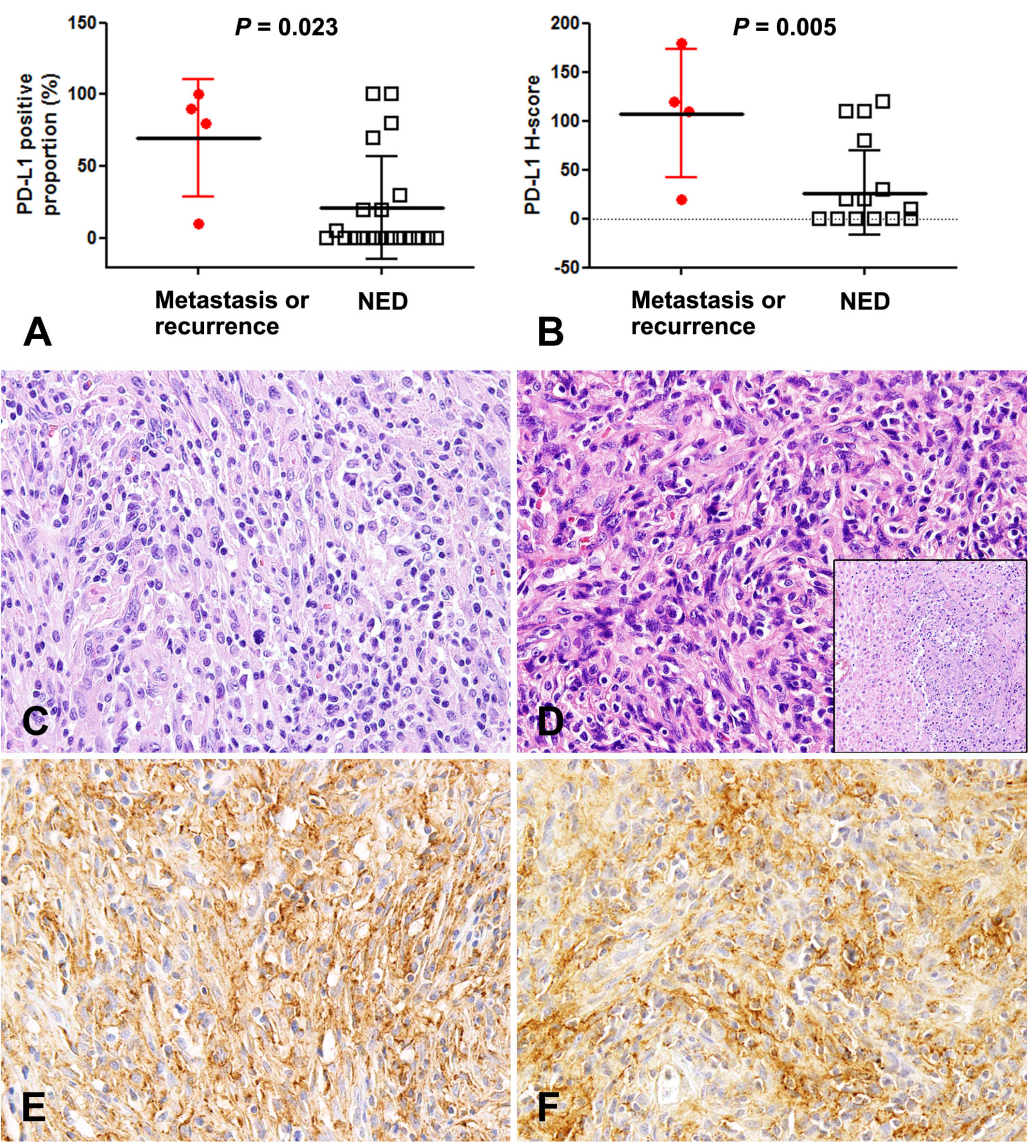
Higher PD-L1 expression in inflammatory myofibroblastic tumors with metastasis and recurrence Inflammatory myofibroblastic tumors (IMTs) with recurrence and metastasis had significantly greater expression of PD-L1 according to proportion **A.** and H-score **B.**. **C.** A primary tumor that occurred in the maxilla of a 49-year-old man (Case 16). Lung metastasis developed 5.3 months after the initial diagnosis. **D.** The metastatic lesion has tumor necrosis (inset) and increased tumor cellularity, with more severe nuclear atypia and prominent nucleoli, compared to the primary tumor. Diffuse expression of PD-L1 in primary **E.** and metastatic IMTs **F.**. NED: no evidence of disease.

There were four available IMTs from two patients, which included two primary IMTs arising in the maxillary sinus, one local recurrent tumor, and one metastatic lung tumor. These IMTs exhibited significant higher PD-L1 proportions and H-scores in the tumor cells (Figure 5). IMTs with necrosis (*n* = 5) exhibited elevated PD-L1 expression (56.0 ± 51.8% *vs*. 15.0 ± 28.4%, *P* = 0.029).

## DISCUSSION

The present study evaluated the clinicopathological characteristics of IMTs according to *ALK* status, and its relationships with PD-L1 expression and CD8+ TILs. We found that patients with metastasis/recurrence exhibited greater PD-L1 expression, and that *ALK*-negative IMTs had more CD8+ TILs, compared to *ALK*-positive tumors.

IMT is now thought to be a true neoplasm with intermediate biologic potential, as *ALK* rearrangement is now observed in approximately 50% of IMTs [2]. In the present study, *ALK* rearrangement was observed in 44.0% of the cases, and *ALK*-positive IMTs occurred in younger patients, compared to *ALK*-negative cases, which is consistent with the findings of previous studies [4, 8, 9]. However, the lungs and the genitourinary tract were the most common sites of *ALK*-positive IMTs in our study, which is slightly different from the findings of previous studies [3, 8, 10]. For example, Western studies have most commonly found *ALK*-positive IMTs in the mesentery and lungs [3, 8], and the abdominal cavity was most common in a Japanese cohort [10]. This difference may be related to the fact that we only examined Korean patients or a small sample size, and further studies are needed to validate our findings.

Some specific histological features have been reported in lung adenocarcinomas with *ALK* or *ROS1* fusions, such as a mucinous cribriform pattern or solid growth pattern with signet ring cells [11, 12]. However, previous studies have failed to detect an association between histological results and molecular alterations in IMTs [4, 8]. In addition, histological features and *ALK* status have generally been reported to have no effect on prognosis [8]. We also did not detect any associations between histology or prognosis and *ALK* status, although *ALK*-positive IMTs tended to have a better prognosis. Yamamoto et al. have reported similar findings [10].

Although most IMTs have a relatively benign clinical course, a subset of patients experience local recurrence and metastasis. In these cases, patients with *ALK*-positive IMTs may benefit from treatment using crizotinib (an ALK inhibitor) [5]. Unlike patients with *ALK*-positive IMTs, patients with advanced *ALK*-negative tumors have no curative treatment options, and receive conventional palliative chemotherapy or radiotherapy. Immune checkpoint inhibitors have recently emerged as a new treatment option for somatic malignancies, including advanced non-small cell lung cancer [13, 14], advanced melanoma [6, 15], and head and neck squamous cell carcinoma [16]. PD-L1 expression is suggested as a predictive biomarker for responsiveness to anti-PD-1/PD-L1 blockade therapy, and is associated with a high mutation burden and neoantigens [17, 18]. Interestingly, dense TILs, especially CD8+ cytotoxic T-cells, can predict treatment response among patients with melanoma who receive anti-PD-1 therapy [19]. TILs are a component of the tumor microenvironment and are thought to be recruited during the immune response to neoantigens that are produced by a tumor [20, 21]. In breast and colon cancers, dense TILs are a predictor of chemotherapy response and favorable outcomes [22, 23]. The US Food and Drug Administration has also recently approved immune checkpoint inhibitors that target CTLA-4 and PD-1.

Teng et al. have classified cancers into four groups based on their TILs and PD-L1 statuses [21]. In that classification, IMTs are considered type I (TIL+ and PD-L1+) or type IV tumors (TIL+ and PD-L1-). As immune checkpoint blockade restores the function of cytotoxic T-cells, the presence of CD8+ TILs and high PD-L1 expression are important factors to consider. Furthermore, IMTs are tumors with intermediate biologic potential, and are expected to have a low mutational burden, compared to highly aggressive carcinomas with a high mutational burden [17, 18]. In the present study, patients with aggressive IMTs exhibited greater PD-L1 expression and did not harbor *ALK, ROS1*, or *RET* rearrangements. Moreover, *ALK*-negative IMTs had more CD8+ TILs and tended to lead to poorer outcomes, compared to *ALK*-positive IMTs. However, only 3 patients in this study experienced metastasis and/or recurrence, and we were unable to adequately evaluate PD-L1 expression, CD8+ TILs, and *ALK* status. Previous studies showed the association between mutation burden and T-cell infiltration [24, 25]. CD8+ TILs reactive to clonal neoantigens were identified in early-stage tumor, and neoantigen specific CD8+T cells appeared to be enhanced by anti-PD-1 therapy [26, 27]. Thus, it can be speculated that high numbers of CD8+ TILs in *ALK*-negative IMTs would imply the presence of more neoantigens, compared to *ALK*-positive IMT. Interestingly, greater PD-L1 expression was observed in IMTs with tumor necrosis and metastasis/recurrence, which were also negative for *ALK* rearrangement. Thus, given that PD-L1 tended to be associated with a poorer prognosis, those cases appear to be potential candidates for immune checkpoint blockade therapy, especially *ALK*-negative IMTs that do not qualify for tyrosine kinase inhibitor treatment.

In the present study, we presumed that PD-L1 expression and CD8+TILs as well as *ALK* status may be associated with biologic behavior of IMT, and expected that immune check point inhibitor might be beneficial to the patients with *ALK*-negative IMTs. However, there are several limitations in the present study. First, the number of included case was too small, as cases were obtained from a single institution, and only three patients experienced metastasis and/or recurrence. It was not adequate for appropriate statistical comparison, and also may be insufficient to conclude that *ALK*-negative IMT takes more aggressive clinical course than *ALK*-positive IMT. Second, there is still a concern that included *ALK*-negative IMTs in this study might belong to unclassified low grade sarcoma or other tumor, although we thoroughly examined included cases with all available ancillary tests and excluded the differential diagnoses. Last but not least, one can have a question whether *ALK*-negative IMTs are tumors of higher mutation burden compared to *ALK*-positive tumors. Since there may be other explanations for T cell infiltration of the tumor, except mutation burden, further studies are needed to determine what mechanism is involved in T cell infiltration of IMT.

In conclusion, we identified distinct clinicopathological characteristics of IMTs according to *ALK* status among Korean patients. We also observed that aggressive IMTs had elevated PD-L1 expression and lacked *ALK* rearrangement, which might be useful for selecting patients for immune checkpoint blockade, although a large-scale study is needed to validate these findings.

## MATERIALS AND METHODS

This retrospective study was performed with approval of the institutional review board of Severance Hospital (4-2015-1081).

### Case selection

Twenty-eight IMTs from 25 patients were retrieved from our center's pathology files (2005-2015). Twenty-five samples were resected specimens and three samples were biopsied specimens. One patient had a renal IMT with lung metastasis. One patient had one recurrent IMT in maxillary sinus and one metastatic IMT in lung.

The diagnosis of IMT was based on the morphological features and pathological criteria from the World Health Organization (WHO) classification of soft tissue tumors, as well as results from immunohistochemistry (IHC) and fluorescent *in situ* hybridization (FISH) [1]. Histologically, IMTs are composed of myofibroblastic and fibroblastic spindle cells with varying proportions of inflammatory infiltrate. Thus, we carefully excluded other possible spindle cell lesions, such as IgG4-related disease, spindle cell carcinoma, and dedifferentiated liposarcoma with IMT-like features [28]. We comprehensively examined the patients’ clinical features and performed IgG4 and IgG IHC to exclude IgG4-related disease in cases with fibrosis and extensive plasma cell infiltration. Spindle cell carcinoma was excluded based on the results of cytokeratin IHC and precise histological examination. In cases of dedifferentiated liposarcoma with IMT-like features, thorough specimen sampling and histological identification of well-differentiated liposarcoma areas were performed.

### Clinicopathological analysis

The following clinicopathological parameters were recorded: age at diagnosis, sex, anatomical location, tumor size, tumor characteristics, treatment modality, date of death, metastasis, recurrence, overall survival (OS), and disease-free survival (DFS). Whole tumor sections from each case were reviewed by two experienced pathologists (YJC and HSS). Based on a previous report [1], the shape of the tumor cells was separately evaluated (spindle/elongated *vs*. plump epithelioid, Figure 1) before assessing nuclear atypia, nucleolar prominence, and the presence of necrosis.

### Tissue microarray

Based on a retrospective review of the hematoxylin and eosin-stained slides, the most appropriate formalin-fixed paraffin-embedded tumor tissue samples were retrieved and representative tumor areas were circled. Two 3.0-mm tissue cores were obtained from the circled area of each paraffin block, and the cores were inserted into 6 × 5 blocks.

### Immunohistochemistry and fluorescent *in situ* hybridization for *ALK*, *ROS1*, and *RET*

IHC was performed using tissue microarray slides and antibodies against anaplastic lymphoma kinase (ALK; D5F3, 1:50; Cell Signaling Technology, Danvers, MA, USA), ROS1 (D4D6, 1:50; Cell Signaling), and RET (C-19, 1:100; Santa Cruz Biotechnology, Santa Cruz, CA, USA). The IHC results were obtained using the OptiView Detection Kit (Ventana Medical System, Tucson, AZ, USA). All cases were reviewed by two experienced pathologists (YJC and HSS). All cases were further analyzed to confirm rearrangement of *ALK*, *ROS1*, and/or *RET* using FISH and a break-apart probe for ALK (Abbott Molecular, Abbot Park, IL, USA), a ROS1 probe (Abbott Molecular), and a RET probe (ZytoVision, Bremerhaven, Germany) [29].

### Immunohistochemistry for PD-L1 and CD8

IHC was performed using whole-tissue slides and antibodies against PD-L1 (SP263, rabbit monoclonal primary antibody; Ventana Medical System) and CD8 (C8/144B, ready-to-use monoclonal mouse antibody, Agilent Technologies, Santa Clara, CA, USA). The IHC results were obtained using the OptiView Detection Kit (Ventana Medical System) and the UltraView Detection Kit (Ventana Medical System), respectively. The PD-L1 IHC results were interpreted according to the methods of previous studies, and positive results were defined as ≥1% of tumor cells with membranous staining [30, 31]. In addition to the proportion of PD-L1 positivity, the H-score was calculated for the PD-L1 staining. This score is calculated by multiplying the intensity value (0, 1, 2, or 3) by the extent of each staining intensity (%). The proportion of intratumoral CD8+ TILs was also recorded [31–33]. Necrotic areas were excluded from the scoring.

### Statistical analysis

Data were reported as number and percentage or mean ± standard deviation. The analyses evaluated PD-L1 expression, CD8+ TILs, clinicopathological parameters, and *ALK* status. The chi-square test and Fisher's exact test were used for categorical parameters, and the *t*-test was used for continuous parameters. OS and DFS were evaluated using the Kaplan-Meier method and the log-rank test. Statistical analyses were performed using IBM SPSS software (version 19.0; IBM Corp., Armonk, NY), and *P*-values of < 0.05 were considered statistically significant.

## SUPPLEMENTARY MATERIALS FIGURES


