# Supplementary material for: PD-L1 expression and CD8+ tumor-infiltrating lymphocytes are associated with ALK rearrangement and clinicopathological features in inflammatory myofibroblastic tumors
Source: Oncotarget. 2017 Sep 15;8(52):89465–74. doi: 10.18632/oncotarget.20948 (PMC5685684; doi:10.18632/oncotarget.20948)
Supplement: Supplementary file 1 [file oncotarget-08-89465-s001.pdf]

PD-L1 expression and CD8+ tumor-infiltrating lymphocytes are associated with *ALK* rearrangement and clinicopathological features in inflammatory myofibroblastic tumors

SUPPLEMENTARY MATERIALS

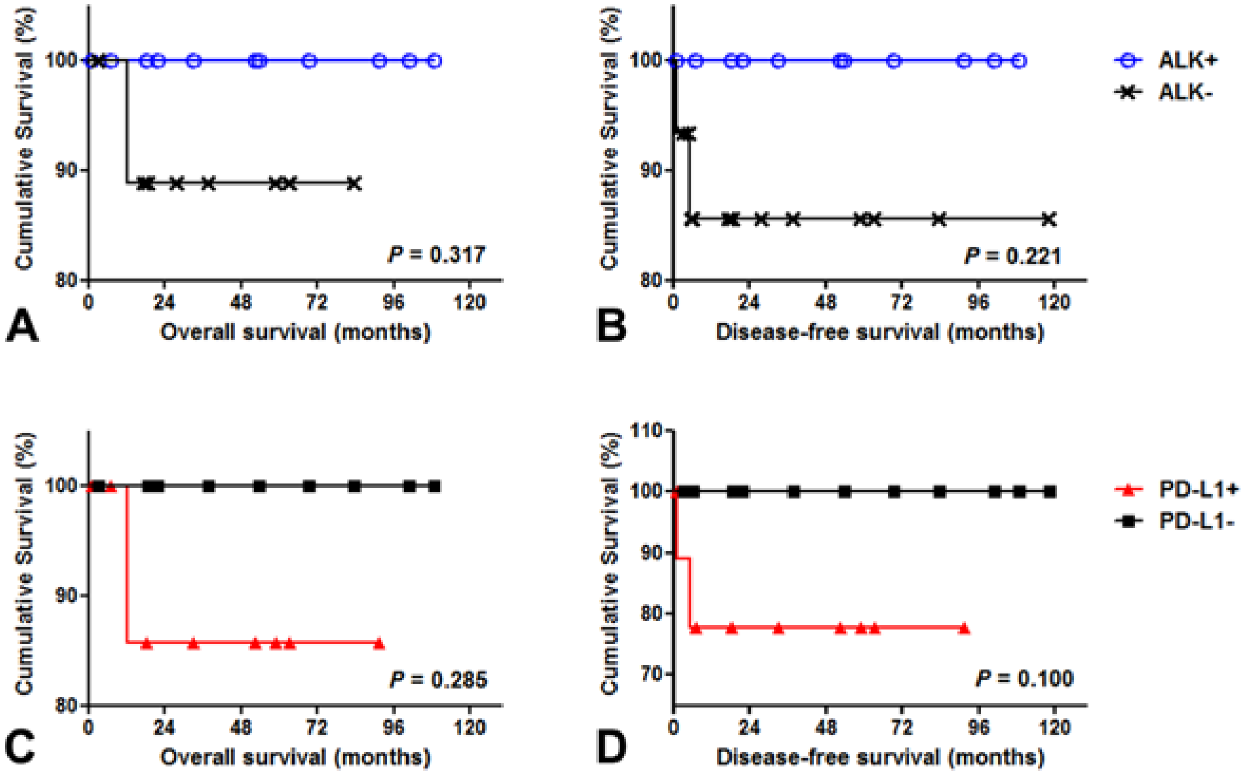

Supplementary Figure 1: Overall survival and disease-free survivals for patients according to *ALK* and PD-L1 status. Although not statistically significant, the survival curves for all patients varied according to *ALK* (A, B) and PD-L1 expression status (C, D). All patients with *ALK*-rearranged inflammatory myofibroblastic tumors (IMTs) were alive at the end of the study (A) and had not experienced metastasis or recurrence (B). PD-L1-positive IMTs had tended to be associated with shorter overall survival (C) and disease-free survival (D), compared to PD-L1-negative IMTs.
